# Regulation of antioxidant mechanisms by *AtDREB1A* improves soil-moisture deficit stress tolerance in transgenic peanut (*Arachis hypogaea* L.)

**DOI:** 10.1371/journal.pone.0216706

**Published:** 2019-05-09

**Authors:** Hiren Bhalani, Radhakrishnan Thankappan, Gyan P. Mishra, Tanmoy Sarkar, Tejas C. Bosamia, Jentilal R. Dobaria

**Affiliations:** 1 Directorate of Groundnut Research, Junagadh, Gujarat, India; 2 Junagadh Agricultural University, Junagadh, Gujarat, India; Estacion Experimental del Zaidin, SPAIN

## Abstract

The present study evaluated the soil-moisture deficit stress tolerance of *AtDREB1A* transgenic peanut lines during reproductive stages using lysimetric system under controlled glasshouse conditions. The antioxidant activities of *AtDREB1A* transgenic lines were measured by biochemical assays. The transgenic peanut lines recorded significantly lower accumulation of malondialdehyde and hydrogen peroxide than the wild-type. Whereas, specific activity of catalase, guaiacol peroxidase, ascorbate peroxidase, glutathione reductase and ascorbic acid were found to be significantly higher in transgenic lines than in the wild-type line under drought stress. The results showed that the transgenic lines expressed lower oxidative damage than wild-type and could protect themselves from the elevated levels of reactive oxygen species under drought stress. This could be attributed to the regulation of various stress-inducible genes by *AtDREB1A* transcription factor. Improved photosynthetic and growth parameters were also recorded in transgenic lines over wild-type under drought stress. Improved physio-biochemical mechanisms in transgenic peanut lines might have resulted in improved growth-related traits as significant correlations were observed between physio-biochemical parameters and growth-related traits under drought stress. The potential target genes of *AtDREB1A* transcription factor in transgenic peanut lines during drought stress were identified, which helped in understanding the molecular mechanisms of DREB-regulated stress responses. The transgenic line D6 reported the best physio-biochemical mechanisms and growth-related parameters under drought stress over other transgenic lines and wild-type, suggesting it may be used to develop high yielding and terminal drought-tolerant peanut varieties.

## Introduction

Peanut (*Arachis hypogaea* L.) is a legume crop that serves as an important oilseed resources. It is also a rich source of dietary vitamin E and phytosterols and ranks third as the source of vegetable protein to human beings [1]. The crop is widely grown in tropical and semi-arid tropical regions that contribute to nearly 90% of the global peanut production [2, 3]. Various abiotic stresses, such as low and high temperatures, soil-moisture deficit or drought stress (DS), salinity stress and heavy metal stress, adversely affect plant growth and productivity, including the overall peanut production [4, 5, 6]. DS is the primary abiotic stress that is more prevalent compared to other abiotic stresses in different parts of the world and limits global agricultural production [7]. Approximately 20% of the land is under DS, at a given point of time across the globe, hence, considered as one of the most important environmental constraints to crop productivity. Annually, peanut productivity incurs a loss of around 6 million tons due to drought alone, across various parts of the world [8]. Harmful effects of DS are manifested by increased membrane permeability, which, in turn, results in altered water relations, mineral nutrition, and plant metabolism [9]. It also has a negative impact on the photosynthetic elements, such as thylakoid electron transport, phosphorylation, and carboxylation, thus affecting the overall yield of the peanut crop [10].

Tolerance to DS is provided by complex physio-biochemical pathways that are regulated by multiple genes [9, 11]. Very less information is available about the QTLs controlling DS tolerance in peanut. Hence, conventional breeding approach is not very successful in the development of drought tolerant peanut varieties [12]. Although the marker-assisted breeding approach has been adopted, but the results are not very encouraging and satisfactory as the DS tolerance is reportedly governed by a few rare alleles present in the existing peanut germplasm [1]. To surmount these limitations, various researchers are now exploiting the transgenic approach to achieve desired improvements in peanut crop toward various abiotic stress tolerances including DS [8, 13].

Development of transgenic (T) peanut lines using various transcription factors (TFs), which regulate the expression of diverse stress-responsive genes, is an effective method to improve DS tolerance [1, 14]. In this regard, dehydration responsive element binding (*DREB*) (belonging to ethylene-responsive element binding factors (ERF) family) has been found to effectively modify the expression of numerous stress-inducible genes, thereby imparting tolerance to DS in peanut [14, 15]. The *DREB*, upon binding to the *DRE/CRT cis*-acting elements of promoter regions of several stress-related genes, induces the expression of these genes and thereby imparting increased tolerance to DS [16]. Heterologus expression of *DREB1* gene in transgenic rice [17, 18], wheat [19], chickpea [20, 21], sweet potato [22] and tomato [23] showed tolerance to DS, salinity and low-temperature stress with improved physio-biochemical and growth-related traits, yield and yield-components.

Overexpression of *AtDREB1A* gene in T peanut (cv. JL 24) provides enhanced tolerance to DS by improving its transpiration efficiency, reducing lipid peroxidation levels and increasing biosynthesis of antioxidant enzymes and proline [12, 15, 24]. In addition, *AtDREB1A* transgenic peanut (cv. JL 24) lines are characterised by improved growth-related traits, such as harvest index, root length, root to shoot ratio and relatively less reduction in pod-yield over wild-type (WT) under DS [25, 26, 27].

The *DREB1A* transgenic peanut (cv. JL 24) lines developed extensive root system upon exposure to terminal DS. Such a root system is more effectively able to trap the available soil-moisture content from its vicinity [28]. Lysimetric system is a preferred method for screening of plants for soil-moisture deficit stress tolerance at the adult stage, as it simulates the field-type conditions and provides the desired space for the plant canopy and roots to grow in controlled conditions. *AtDREB1A* transgenic peanut lines were developed, in our laboratory, with a genetic background of GG20 cultivar, which is high yielding, bold seeded, ruling and very popular in western parts of India [8, 10, 15]. In the present study, *AtDREB1A* transgenic peanut (cv. GG20) lines were evaluated for their performance under progressive soil-moisture deficit stress in lysimeters system, simulating the field-like conditions.

Further, various characteristics of *AtDREB1A* transgenic lines were studied including their performance at reproductive growth stages, such as flowering, pegging and pod-formation, for various physio-biochemical and growth parameters along with yield components. Moreover, this study is the maiden attempt to understand the expression pattern of various stress-inducible genes involved in DS signalling pathways and are regulated by *AtDREB1A* gene in T peanut lines grown under lysimetric system. It was hypothesised that the better activation of various antioxidant enzymatic systems in the *AtDREB1A* transgenic peanut lines over WT, helps to improve the oxidative stress caused by the DS.

## Materials and methods

### Plant material and creation of soil-moisture deficit stress in the lysimeter

Eight *AtDREB1A* transgenic peanut (cv. GG20) lines (D1 to D8) that were earlier developed and confirmed in our laboratory were used for this study. These lines are previously characterised at seedling and flowering stages (T_2_) in hydroponic solution and soil-containing pots, respectively under DS and salinity stresses [8, 15]. These T peanut lines (T_3_) were used for the evaluation of DS response through soil dry-down experiments using the lysimeter at adult plant stage.

A total of 18 plants (45 days old) of each T line along with 3 WT were grown individually in the lysimeter which consisted of PVC tubes (1.0 m long and 20.0 cm wide), containing soil mixture (42.0 kg) having soil, sand and farmyard manure (FYM) in the ratio 1:2:0.1. All experiments were performed at the ICAR-Directorate of Groundnut Research, Gujarat, India in a glasshouse with 16 hours of light at 35±2°C for 120 days. At day 45 after germination (DAG), all 8 T lines were grouped into two treatments, namely well-watered (WW) and drought-stressed (DS) with three replications. First, the soils of both WW and DS were irrigated till field-capacity [29], followed by covering of soil surface with plastic beads (500 g per lysimeter) to prevent the evaporation. The mass of each lysimeter was then measured at a 3-day interval from 09:00 to 10:00 am throughout the experiment.

The WW plants were supplemented with the same volume of water that was lost via transpiration to maintain the water level at nearly 80% of its field capacity. However, the DS condition was created by withholding the irrigation until the appearance of wilting symptoms. For the physio-biochemical and molecular analysis, the leaf samples were collected at 0 (flowering stage/control condition/initial stage), 10 (pegging stage) and 25 (pod formation stage) days of DS imposition from both WT and T lines. During termination of DS experiment, stressed plants were also irrigated using 200 mL of water for 3 d for 24 h for their recovery.

### DNA extraction and homozygosity confirmation of transgenics

The polymerase chain reaction (PCR)-confirmed homozygous T_2_ plants [8] were grown in the glasshouse to obtain the T_3_ generation. Total genomic DNA was isolated from the young leaves of T_3_ and WT plants using the cetyltrimethylammonium bromide (CTAB) method [30]. Following this, all T plants were screened by PCR using *AtDREB1A* transgene-specific primers.

### Physiological and biochemical analysis

#### Soil moisture content and hydrogen peroxide (H_2_O_2_) measurement

Soil samples were collected from 30 to 45 cm of depth of lysimeters using an auger screw and fresh mass (FM) was measured. The soil samples were then dried at 72°C for 2-days, following which dry mass (DM) was measured [31]. The soil moisture content was estimated from each lysimeter at various plant growth stages, i.e., flowering, pegging and pod formation using the formula: (FM−DM)/DM × 100.

For H_2_O_2_ estimation, approximately 1.0 g of leaf tissue was macerated in 10 mL of chilled acetone and filtered using Whatman no. 1 paper. To this 5 mL extract, 2 mL of titanium reagent and 2.5 mL of concentrated ammonium solution were added. The mixture was then centrifuged at 10,000×g for 5 min. The peroxide–titanium complex so obtained was then dissolved in 2M H_2_SO_4_ (5 mL) and absorbance was measured against the blank at 415 nm [32]. The H_2_O_2_ content was determined by plotting a standard curve with known concentrations of H_2_O_2_.

#### Relative water content (RWC), chlorophyll content and malondialdehyde content measurement

The RWC was calculated in the leaf discs (1 cm in diameter) taken from T and WT plants. The initial mass was measured as FM and subsequently, these discs were hydrated for 8 h in a Petri plate and weighed to get the turgid mass (TM). These discs were then dried at 80°C for 72 hours in a hot-air oven and weighed till a constant DM was reached. The RWC was measured using the formula: (TM–DM)/(FM–DM) × 100 [33].

The chlorophyll content was measured (*n* = 3) using a chlorophyll meter (SPAD-502 Plus, Konica Minolta, Japan) from the peanut leaflets by measuring the absorbance in the red and near-infrared regions. The lipid peroxidation level was measured using thiobarbituric acid (TBA) method and expressed as malondialdehyde (MDA) content [34]. One gram of leaf tissue was first crushed in 10 mL of trichloroacetic acid (TCA, 0.1%; m/v) and centrifuged at 15,000×g for 15 min. To 1 mL of supernatant, 4 mL of TBA reagent (0.5%, m/v TBA in 20%, w/v TCA) was added and mixture was heated (95°C for 30 minutes) and the reaction was blocked by placing it on ice. The mixture was again centrifuged at 10,000 ×g for 10 min and absorbance was measured (532 nm) and the amount of MDA was calculated using the extinction coefficient of 155 mM^–1^cm^–1^ and expressed as μmol g^–1^ FW.

#### Estimation of ascorbic acid

For the ascorbic acid (AsA) estimation, 250 mg of the leaf was ground in 10.0 mL of TCA (6%, m/v). The extract (2.0 mL) was mixed thoroughly after adding 1.0 mL of dinitrophenyl hydrazine (2%, m/v) and 1 drop of thiourea solution. Afterward, it was boiled for 15 min, cooled to room temperature and 5.0 mL of H_2_SO_4_ (80%, v/v) was added to obtain hydrazone complex. The absorbance was measured at 530 nm, and the AsA content was derived from the standard curve prepared through known AsA concentrations [35].

#### Antioxidant enzymatic activity

For the estimation of catalase (CAT), guaiacol peroxidase (POD), ascorbate peroxidase (APX), and glutathione reductase (GR) enzyme extracts, 1.0 g of leaf was macerated in liquid nitrogen. To this, 10 mL of ice-cold 100 mM potassium phosphate extraction buffer (pH 7.5) containing 5.0 mM EDTA was added. For the estimation of APX, ascorbic acid (1.0 mM) was mixed with extraction buffer and the homogenate was centrifuged at 15,000 ×g for 20 min. The supernatant was stored as aliquots at −20°C till final analysis.

For CAT estimation, the reaction was completed in a 3.0-mL mixture containing 50 mM phosphate buffer (pH 7.0), 12.5 mM H_2_O_2_ and 50 μL extract. Decomposition of H_2_O_2_ was measured as a reduction in the absorbance at 240 nm for 2.0 min at 10 s of the time gap. The CAT activity was presented as μmol of H_2_O_2_ reduced mg^–1^protein min^–1^ [36].

For POD estimation, the reaction was completed in a 3.0-mL mixture containing 50 mM potassium phosphate buffer (pH 6.1), 16 mM guaiacol, 2 mM H_2_O_2_ and 25 μL enzyme extract. After the addition of 2 mM H_2_O_2_, the oxidation of guaiacol to tetraguaiacol was monitored by measuring the absorbance at 470 nm for 1 min at a 15-second interval. The POD activity was estimated using tetraguaiacol (26.6 mM^–1^cm^–1^) extinction coefficient and presented as mmol tetraguaiacol formed mg^–1^ protein min^–1^ [37].

For APX activity, the reaction was performed in a 3.0-mL solution having potassium phosphate buffer (50 mM; pH 7.0), EDTA (0.1 mM), ascorbate (0.5 mM), H_2_O_2_ (0.1 mM) and enzyme extract (0.1 mL). The oxidation rate of ascorbate was calculated by measuring the reduction in absorbance at 290 nm for 2 minutes at a 10-second interval. Further, the APX activity was derived by estimating the reduction in AsA content through the standard curve [38].

For GR activity, the reaction was performed in a 3.0-mL mixture containing potassium phosphate buffer (100 mM; pH 7.5), 5,5-dithiobis-2-nitrobenzoic acid (0.5 mM; DTNB), NADPH (66 μM), oxidised glutathione (GSSG; 0.66 mM) and extract (0.1 mL). The increase in absorbance at 412 nm was measured for 2 min at the 10-s interval and GR activity was expressed as μmol GSSG reduced mg^–1^ protein min^–1^ [39]. In all enzymatic preparations, the protein content was measured through Lowry’s method using bovine serum albumin (BSA) as the standard [40].

### Yield and growth components under well-watered and drought-stressed conditions

Various growth parameters including root length, shoot length, pod mass, and root volume were measured in WT and T lines at the time of maturity (day 120). Subsequently, the plants were dried in the oven (70°C for 72 h) and the traits such as root dry mass and shoot dry mass were measured [8].

### Isolation of RNA and reverse transcription polymerase chain reaction (RT-PCR)

Total RNA was extracted from the leaves of T lines D3, D6, and WT that were collected at day 25 of DS imposition. The lysimeter recorded the soil-moisture content between 5.1 and 7.59% at the time of leaf collection in WT and T lines under DS. The T lines D3 and D6 showed the best performance under DS in terms of improved physio-biochemical, growth and yield parameters. The same parameters determined the stress-responsive regulatory mechanism of *AtDREB1A* gene in the T peanut under DS. Total RNA was isolated from the young leaves of all eight T lines along with the WT from both WW and the DS treatment [8]. The cDNAs were synthesised using the first-strand cDNA synthesis kit (Fermentas, United States). These were then reverse transcribed from three independent biological replicates. Expression levels were measured using a StepOne Real-Time PCR system (Applied Biosystems, United States) and SYBR Green qPCR Master Mix (Qiagen, GmbH, Germany). The PCR amplification was performed in a reaction mixture (20 μL) containing master mix (10 μL), forward and reverse primers (0.8 μL; 10 μM), cDNA (2 μL) and double-distilled water (ddH_2_O, 6.4 μL). The PCR amplification programme included a pre-denaturation step at 95°C for 5 min; 40 cycles of 95°C for 10 s and 60°C for 30 s; and a melting curve step at 95°C for 15 s, 60°C for 1 min and 95°C for 15 s. The 18S RNA was used as the reference, and 2^−ΔΔCT^ methods were applied to calculate relative expression levels [41,42].

The peanut ESTs (expressed sequence tag), assembled into unigenes by Bosamia et al. [43], were used to design the primers (S1 **Table**) for real-time analysis. Based on the functional annotation, the unigenes associated with plant’s DS response were selected for deciphering the regulatory function of *AtDREB1A* regulons on the expression of selected downstream stress-inducible genes through quantitative real-time PCR (qRT-PCR).

### Statistical analysis

Analysis of variance (ANOVA) and Tukey’s multiple range tests (LSD *p* ≤ 0.05) were used to calculate the significance of variation between the WT and T lines. All the assays were performed in triplicate and the data were analysed using software DSAASTAT version 1.1 [44]. The correlation coefficient of all physiological and biological data was derived using the PAST (Paleontological Statistics, version 1.89).

## Results and discussion

### Molecular characterisation

**Fig 1:**
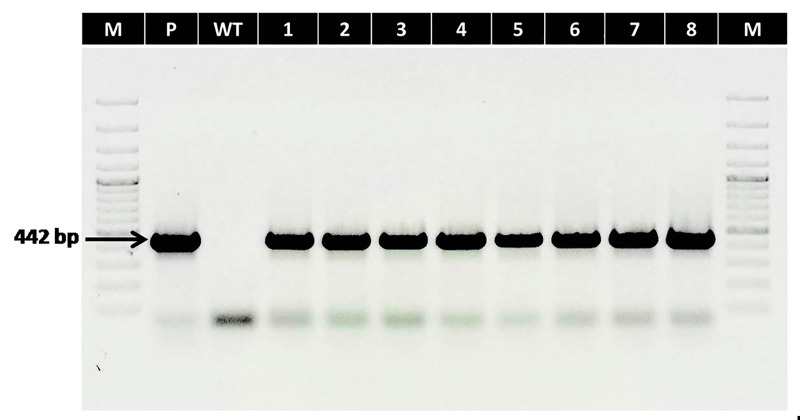
PCR screening of transgenic lines (T_3_) using *AtDREB1A* transgene-specific primers with expected amplification size of 442 bp. Lanes–M: 100bp marker; P: Plasmid positive control (pCAMBIA2300 having *AtDREB1A* gene); WT: Non-transformed control; 1–8: Transgenic lines (D1-D8).

Fifty-four homozygous T plants (T_3_) derived from the confirmed T_2_ plants were selected for further study. These plants were again confirmed for homozygosity via *AtDREB1A* transgene-specific PCR assay (**Fig 1**).

### Physiological and biochemical attributes

#### Soil moisture content

**Fig 2:**
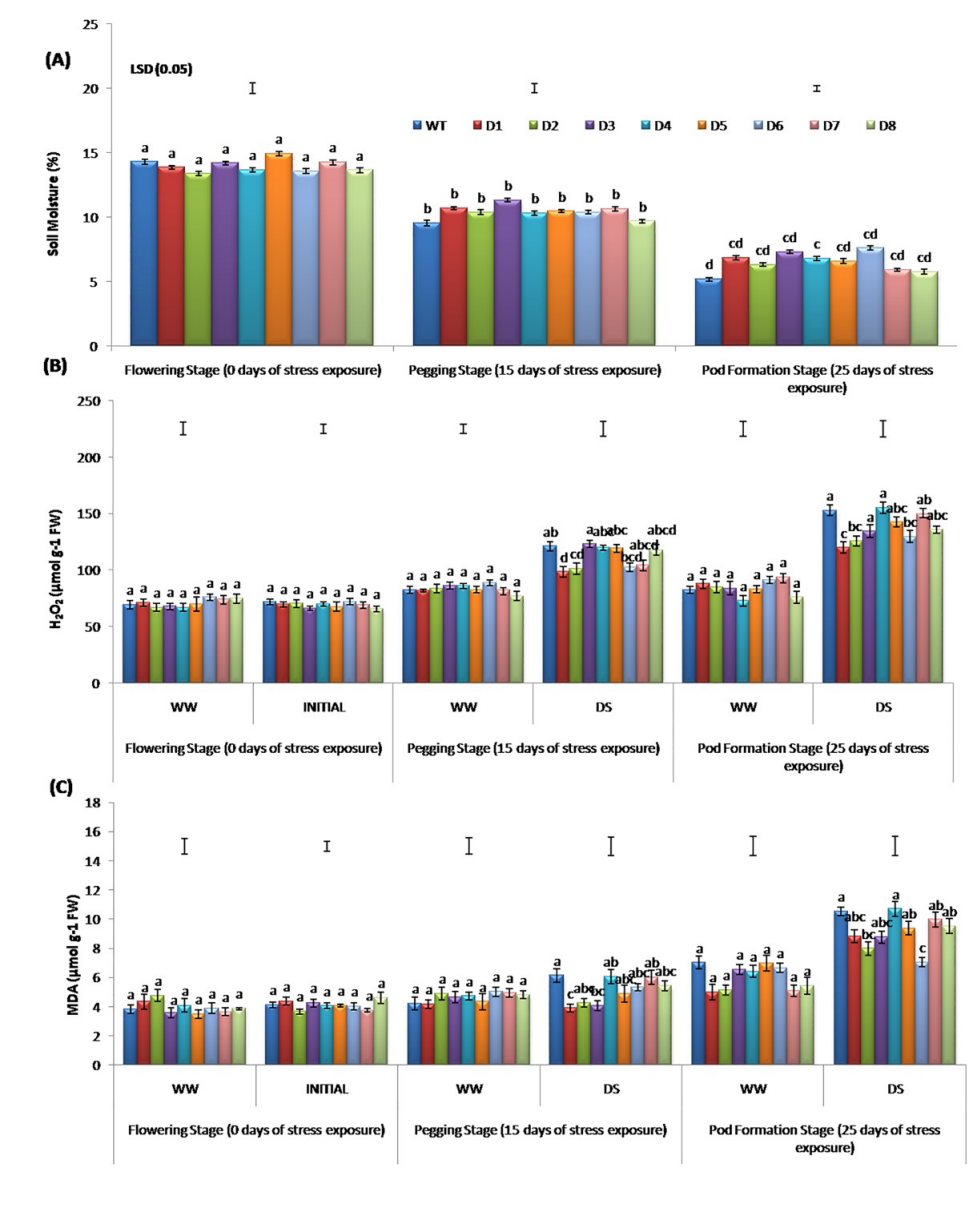
Changes in the (A). Soil moisture content, (B). H_2_O_2_ content, (C). MDA contents of WT and T lines at 0, 15 and 25 days after stress imposition in the lysimeter.

Significant variation in the soil-moisture content of WT and T lines was recorded at day 25 after DS imposition. The control plants were irrigated at regular time intervals. However, with the progression of DS, a gradual reduction in soil-moisture content was observed in both WT and T lines. We also observed a significant difference in the soil-moisture content from a lower 5% in WT to a maximum of 7.59% in the D6 transgenic line (**Fig 2A**). Soil-moisture content was reduced by 2.75 fold in WT, while in D3 line the reduction was 1.78 fold. Further, the T lines produced more fine roots, which could have helped in mining more moisture in the root zone. Also, the low transpiration rate of the T lines also helped in efficient water conservation over WT [8, 15, 45].

#### Estimation of hydrogen peroxide and malondialdehyde content

Among the eight T lines, D1, D2 and D6 reported significantly lower H_2_O_2_ accumulation when compared to WT at day 15 and 25 of DS, thus suggesting D1, D2 and D6 to have better DS tolerance. Further, most T lines accumulated a lower quantity of H_2_O_2_ compared to WT under DS (**Fig 2B**) as also reported in *Mentha arvensis* under cadmium induced oxidative stress [46]. Similarly, lower level of H_2_O_2_ was also recorded in *SbDREB2A-*tobacco than the WT under polyethylene glycol (PEG) induced dehydration stress [47].

Abiotic stress causes peroxidation of cell membrane lipids, resulting in a significant increase in MDA content, which is indicative of enhanced toxic oxygen species synthesis [48]. The MDA levels serve as an indicator for lipid peroxidation which results from the oxidative stress and generation of ROS [49]. The MDA content in T lines and WT did not change significantly prior to the stress, but a significant difference was observed between T peanut lines and WT at day 15 of DS. The maximum MDA content was recorded at day 25 of DS (**Fig 2C**) and all T lines except D4, D7 and D8 accumulated lower MDA content than WT. Similarly, significantly lower MDA accumulation was noticed in *OsAP21 Arabidopsis* than its WT under both DS and salinity stress conditions [50]. The *AtDREB1A* gene in the T peanut was found conferring enhanced ability to eliminate oxygen and H_2_O_2_, thereby inhibiting ROS accumulation. This, in turn, protected the T plants from lipid peroxidation and oxidative damage under DS. Therefore, when compared to T line, more MDA was found to be accumulated in WT under DS. These results are in agreement with the previous reports on pepper [51] and Thomson navel orange [52].

#### Relative water content and chlorophyll colour index

**Fig 3:**
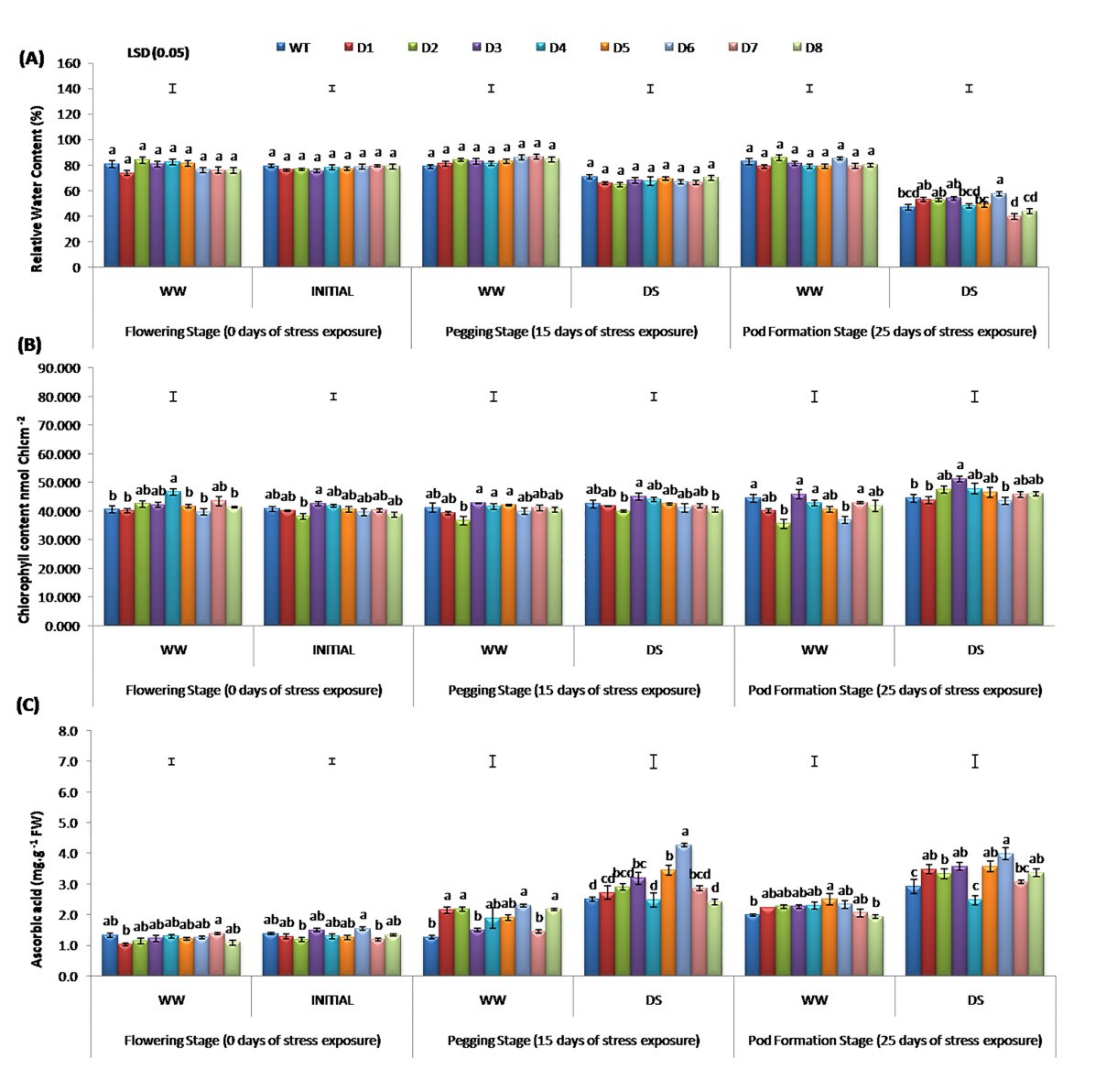
Changes in the (A). RWC, (B). Chlorophyll colour index in leaves, (C). Ascorbic acid content of WT and T lines at 0, 15 and 25 days after stress imposition.

Both chlorophyll content and RWC of plant are generally considered as indicators for measuring the degree of DS tolerance. The RWC of any plant under DS reflects the metabolic activities of its tissues [13]. A considerably higher RWC and chlorophyll colour index (CCI) was recorded in T lines D6 and D3, respectively, over WT lines under DS (**Fig 3A and 3B**). It was observed that under WW conditions, the RWC was almost similar (*p* ≤ 0.05) in both T lines and WT. The chlorophyll content decreased in T lines and WT with an increase in exposure to DS; however, it was considerably more in T lines (except D1 and D6), over WT after 25 days of DS (**Fig 3B**). Moreover, the decrease in RWC was not significant in control samples, and also at day 15 of DS imposition in both T lines and WT. After 25 days of DS, WT reported 47.1% RWC, whereas the content varied from a low of 39.8% in D7 to a high of 57.6% in D6 line (**Fig 3A**). This difference could be attributed to the enhanced membrane integrity that helps to control water efflux and retain water more efficiently under DS condition [53]. The T lines containing *AtDREB1A* gene might also shows improved osmotic adjustment, resulting in higher water retention capacity and minimised rate of water loss compared to WT, as reported in an earlier study [53].

Another feature that was affected under DS is the chlorophyll content of leaves (**Fig 3B**) and the reduction is CCI is attributed to the formation of ROS [54]. The photosynthetic performance of *AtDREB1A* expressing T peanut plants was analysed under WW and DS conditions. A progressive increase in soil-moisture deficit stress has led to the further reduction in CCI values in both WT and T lines. However, the rate of reduction was recorded to be higher in WT compared to T lines (except D3 and D6). Thus, DS has led to a significant increase in CCI in T lines over WT (**Fig 3B**). Here, at days 15 and 25 after DS imposition, when compared to WT, the T line D3 had reported a significantly higher SPAD value (24.7%). Moreover, the same line demonstrated significantly enhanced CCI under both DS and WW conditions. Similarly, Sarkar et al. [8] observed better leaf water and chlorophyll retention capacity in T lines (D3 and D6) than WT under DS condition.

A significant reduction in the loss of chlorophyll content in T lines clearly indicated that the photosynthetic apparatus is safeguarded from the lethal effects of soil-moisture deficit stress. It can be stated that the improved chlorophyll maintenance in D3 line over WT and other T lines under DS might have resulted from increased biosynthesis of osmolytes, such as proline, as reported earlier [8]. Proline helps to retain membrane stability and chlorophyll content owing to its osmoprotective nature [55]. Also, it is known to be associated with antioxidant mechanisms to overcome the harmful effects of multiple stresses [15, 55].

#### Antioxidant metabolites and antioxidant enzyme activities

The T peanut lines showed a significantly higher antioxidant enzyme activity than WT when exposed to DS. Abiotic stress leads to altered cellular homeostasis and enhanced ROS synthesis in plants. The ROS, in turn, disturbs the cell membrane properties, causing oxidative damage to lipids, proteins, chlorophylls and nucleic acids [56, 57]. Plants, therefore, should have mechanisms to counter and minimise the effects of DS and evade the build-up of free oxygen radicals when encounters various stresses [58]. The antioxidant enzymatic detoxification mechanism present in the plants involves different ROS scavenging enzymes, such as CAT, APX, POD and GR, which are part of the antioxidative defence system [6, 14, 59]. The components of the antioxidant defence systems include low-molecular mass molecules that mainly consist of non-enzymatic antioxidants (e.g. ascorbic acid, glutathione) and antioxidants enzymes such as CAT, GR and APX. For instance, these antioxidants are part of the Halliwell–Asada and ascorbate–glutathione cycles that function to increase the total antioxidant activity under various stresses including DS [46, 56, 58].

Ascorbic acid (AsA) or Vitamin C is a secondary metabolite that acts as a key player in the detoxification of ROS [60]. It is a component of various enzymatic and non-enzymatic antioxidant defence mechanisms, thereby contributing to ROS neutralisation and better photosynthetic rate, transpiration rate, and oxidative defence potential [59]. Moreover, AsA activity is also used as a reference in various studies related to stress tolerance in plants [61]. Increase in AsA content during DS is indicative of improved growth and enhanced activities of other antioxidant enzymes, such as SOD, POD and CAT [24, 62]. When compared with WT, we found significantly more AsA content in the leaf tissues of DS exposed *AtDREB1A* peanut lines (**Fig 3C**).

The AsA content in the leaf tissue of D6 line was higher at day 15 and 25 of DS (**Fig 3C**). Moreover, AsA was accumulated at significantly higher levels in T and WT plants at day 25 of DS over control conditions, except for D1 line (**Fig 3C**). However, variable results of D4 line could be due to the poor expression of *AtDREB1A*, which is responsible for its uneven performance. This phenomenon is also accounted in other crops such as *mtlD* peanut [63] and *DHAR* tomato [64] under salinity stress.

**Fig 4:**
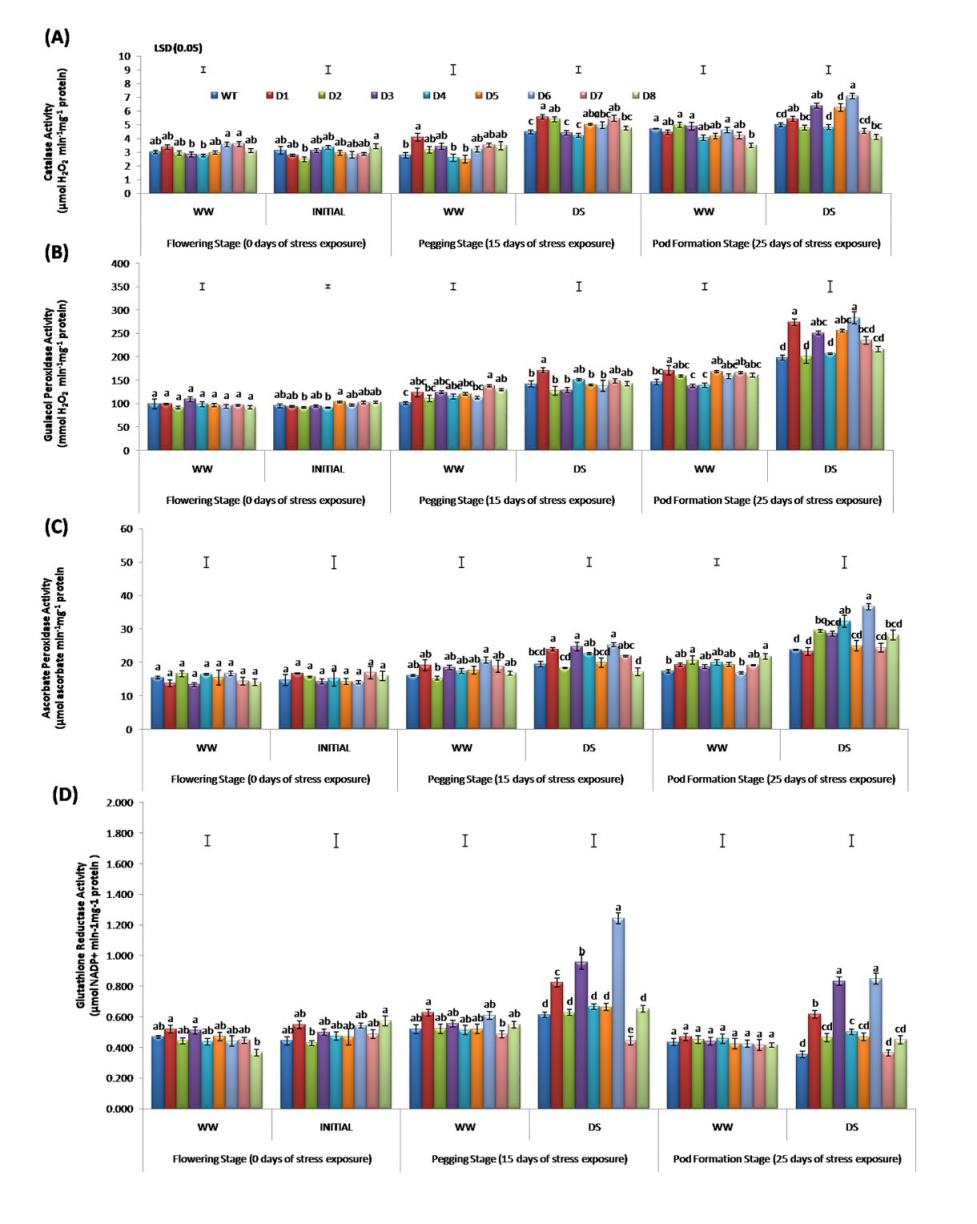
Comparison of various antioxidant enzymatic activities including (A) Catalase, (B) Guaiacol peroxidase, (C) Ascorbate peroxidase and (D) Glutathione reductase in WT and T lines at 0, 15 and 25 days after stress imposition.

The CAT acts as a dismutase that converts H_2_O_2_ into H_2_O and O_2_, whereas POD decomposes H_2_O_2_ by oxidation of co-substrates including phenolic compounds and/or antioxidants [65]. An increase in CAT activity was measured at day 15 of DS in both T and WT peanut plants, followed by a gradual increase at day 25 of DS (**Fig 4A**). At day 25 of DS, the peanut lines D1, D3, D5 and D6 showed significantly higher activities of CAT, POD, APX and GR over WT (**Fig 4B**–4**D**). The specific activity of POD increased in both T lines and WT with an increase in DS at day 25 of DS. A wide variability was recorded among T lines for POD activity and a lower activity was recorded in various T lines except D1, at day 15 of DS, whereas T lines D1, D3, D5 and D6 showed an increased activity at day 25 of DS (**Fig 4B**).

With increasing DS duration, a significant increase in GR activity was noted in T and WT lines, with maximum value quantified at day 15 of DS. GR activity increased by 1.22, 2.03 and 2.38 fold in D6 compared to WT under control conditions at day 15 and 25 of DS, respectively (**Fig 4C**). The POD activity increased by 1.76 fold at day 25 of DS, this activity was recorded at par at day 15 and 25 of DS. Some of the T lines exhibited POD and GR activities at par with the WT at day 15 and 25 of DS. In general, APX and CAT activity in T lines was in the order of D6 > D3; these were significantly higher than the WT.

A significant boost in the APX and GR activities was observed in T peanut lines over WT, with increasing DS (**Fig 4B**). However, at 25 day of DS, there was a decrease in GR activity. Similar to many other parameters, T lines D3 and D6 also expressed better APX and GR activities over other T lines. Enhanced APX and GR activities were also observed in different T peanuts expressing *mtlD* [63] and *AtDREB1A* [24] genes and also showing tolerance to various abiotic stresses.

APX is associated with the ascorbate-glutathione antioxidant cycle that is involved in the scavenging of H_2_O_2_ [66]. However, GR counters the oxidative stress by creating a reduced state of glutathione, thereby maintaining the AsA pool [62]. Under WW conditions, the GR activity in T lines was found to be significantly different than the WT (**Fig 4D**). The main function of GR is to convert the oxidised glutathione disulfide (GSSG) to glutathione (GSH) using NADPH as a cofactor [67]. Different H_2_O_2_ levels in the cell influence the GSH:GSSG ratio; moreover, the interaction between H_2_O_2_/GSH/GR redox system assists in managing various abiotic stresses in the plants [68]. Upon imposition of 25 days of DS, we noted a significant boost in the GR activity in the T lines over WT peanut lines, which could be the result of a change in gene expression regulating its synthesis. These results indicated that the T peanut lines can better tolerate the DS over WT through the abundant synthesis of various antioxidant enzymes.

The antioxidant enzymes act as ROS quenchers and their relative content determines the plants’ sensitivity to lipid peroxidation [69]. The POD activity in *Arabidopsis* accounts for nearly 50% of H_2_O_2_ production during oxidative burst. In addition to alleviating H_2_O_2_, it also functions in cell growth and lignin formation by involving a number of substrates [70]. Therefore, POD alone cannot be considered the sole player in plant defence under stress as its functions are considerably different from that of APX and CAT. Present results indicated a significantly high activity of APX, CAT and POD on day 25 of DS (**Fig 4A–4C**). Antioxidant enzymatic activity is a powerful indicator of DS whose accumulation is considered vital to prevent the dehydration of plant cells by balancing the osmotic strength of cytoplasm with the changing environmental conditions [58].

Formation of ROS is considered a threat to any cell as it may cause lipid peroxidation and membrane injury along with damage to the proteins and nucleic acids [6, 71]. These are formed either as a by-product of various metabolic pathways or through the leakage of electrons from the electron transport system [14, 57]. The innate defence system in plants reacts with oxygen to keep the ROS at a lower level. Also, plants possess antioxidant enzymes to quench the ROS, thereby keeping their levels low [69, 71]. SOD converts the superoxide radical to H_2_O_2_, which is then reduced to water and oxygen either by APX through the ascorbate-glutathione cycle or by POD and CAT in the cytoplasm or other cell compartments [72].

With increasing DS, a significant increase in specific antioxidant enzymatic activities was recorded in both WT and T lines. This in turn, resulted in an efficient detoxification of superoxide radicals and lesser oxidative stress in T peanut lines over WT. On the similar note, improved antioxidant mechanisms showed better survival ability in *AtDREB1A* tomato under DS [56].

### Yield and growth components under well-watered and drought stress conditions

**Fig 5:**
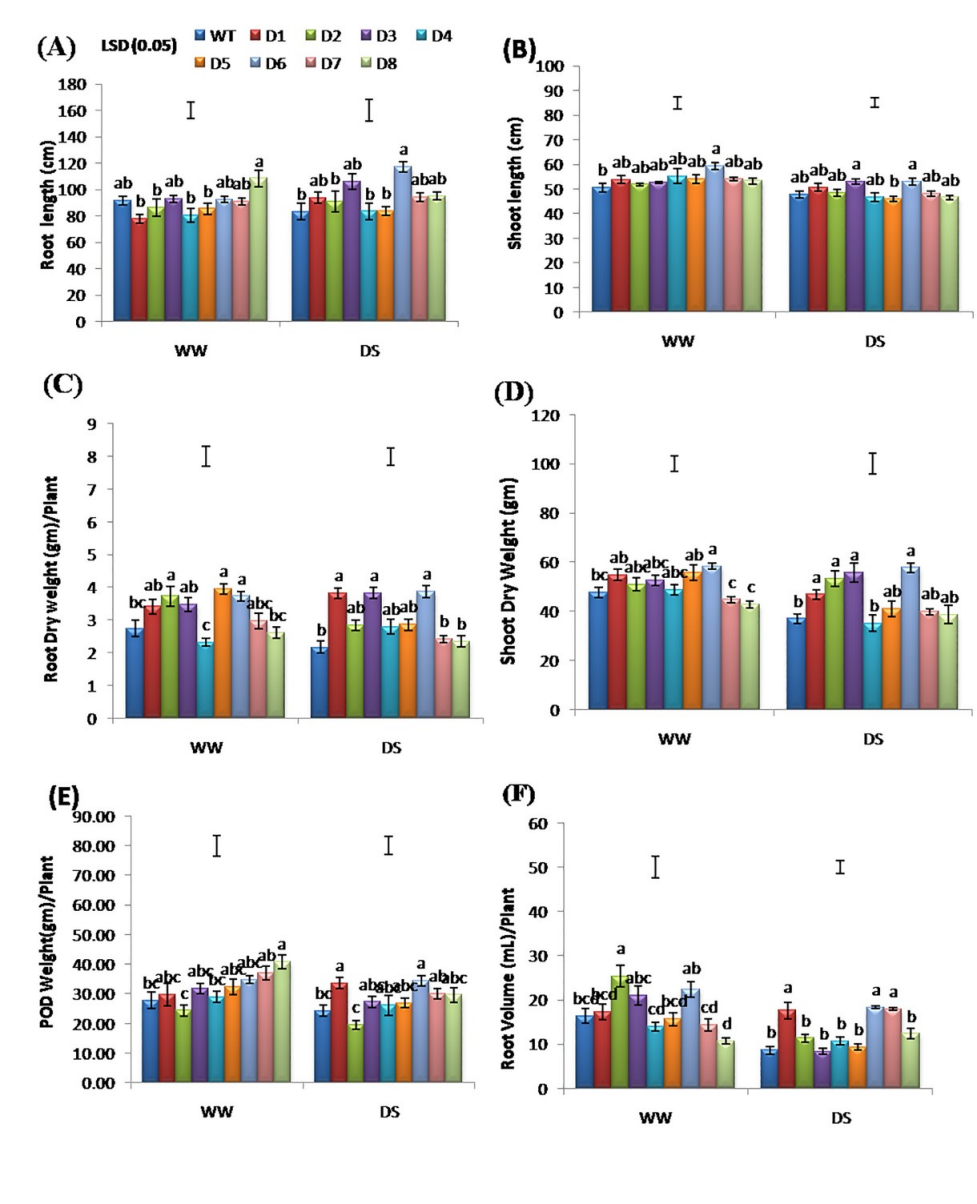
Comparison of various growth parameters in WT and T lines under DS (after 25 days of DS imposition) and WW conditions. Where (A) Root-length, (B) Shoot-length, (C) Root-mass, (D) Shoot-mass, (E) Pod-mass, (F) Root-volume, from WT (GG 20) and eight independent T lines (D1-D8) after harvesting (120 day old plants with maturity).

Considering that the peanut yield gets significantly reduced under DS [2], reports of better growth parameters of various peanut T lines over-expressing *AtDREB1A* [15] and *mtld* [5, 63] genes under various abiotic stresses are of great significance. T lines grown under DS was observed performing better than the WT in terms of various growth parameters. For instance, the shoot length was significantly more in T over WT plants, particularly in the D6 line, which showed 10.80% and 17.10% increase in the length under DS and WW conditions, respectively (**Fig 5B**). Moreover, for root length, D6 line showed 40.96% more growth over WT under DS. The T line D8 showed 18.61% higher root length when compared to WT in WW conditions (**Fig 5B**). Also, the T lines D3 and D6 showed an overall superior performance over WT under DS. Other T lines were found at par with WT for shoot length and root length. Under DS, the root mass and shoot mass of WT were found to be significantly less than the T lines, similar to that reported for *OsSNAC1* transgenic cotton [45].

A significantly better root dry mass was recorded in the T lines D3, D6 and D1 than WT (**Fig 5C**), indicating efficient root architecture for absorbing more water from the deeper soil layers as also reported by Jagana et al. [26] in peanut. Similarly, the shoot dry mass of WT was found significantly lower than the T lines D2, D3, D6 and D1 (**Fig 5C** and **5D**). We reported a notable increase in pod mass in all T lines, except D2 over WT (**Fig 5E**). Whereas, significantly increased root volume was observed in T lines D6, D1 and D8 over WT (**Fig 5F**). The root volume in other the T lines D4, D2, D5, D3 and D8 was found at par with those of WT (**Fig 5F**). Thus, the present findings validated the outcomes of an earlier study where T lines D3 and D6 showed better expression than WT in terms of pod yield and growth-related parameters under DS [8].

### Correlation analysis of physio-biochemical and growth-related parameters

Linear correlation analysis identified the relationship among key biochemical, physiological and growth parameters under DS at pod formation stage of peanut plant (**Table 1**). The antioxidant enzymes showed significant correlations among each other (strong correlation between APX and CAT, *r* = 0.843**; APX and GR, *r* = 0.862**; GR and CAT, *r* = 0.835**; moderate correlation between APX and POD, *r* = 0.758**; POD and GR = 0.768**).

**Table 1:** Correlation between key growth and physio-biochemical parameters at day 25 of DS (pod formation stage).

	GR	CAT	APX	POD	AsA	MDA	H_2_O_2_	RWC	PDM	RL	SL	RDM	SDM
**GR**	1												
**CAT**	0.835**	1											
**APX**	0.862**	0.843**	1										
**POD**	0.768**	0.859**	0.758**	1									
**AsA**	0.668**	0.778**	0.729**	0.803**	1								
**MDA**	-0.626**	-0.691**	-0.710**	-0.645**	-0.770**	1							
**H2O2**	-0.415*	-0.690**	-0.633**	-0.705**	-0.680**	0.575**	1						
**RWC**	0.735**	0.753**	0.701**	0.580**	0.553**	-0.654**	-0.432*	1					
**PDM**	0.414*	0.310	0.281	0.442*	0.388*	-0.182	-0.172	0.078	1				
**RL**	0.712**	0.705**	0.670**	0.646**	0.806**	-0.648**	-0.331	0.522**	0.470*	1			
**SL**	0.352	0.445*	0.293	0.424*	0.372	-0.292	-0.326	0.342	0.055	0.375	1		
**RDM**	0.246	0.243	0.184	0.187	0.250	0.060	-0.062	-0.045	0.658**	0.453**	0.274	1	
**SDM**	0.097	0.328	0.109	0.220	0.384*	-0.487*	-0.418*	0.367	-0.127	0.302	0.415*	0.121	1

Correlation between key growth and physio-biochemical parameters, average values of eight T lines along with WT were used for correlation analysis. Where: GR- glutathione reductase, CAT- catalase, APX- ascorbate peroxidase, POD- guaiacol peroxidase, AsA- Ascorbate, MDA- malondialdehyde, H_2_O_2_- hydrogen peroxide, RWC- relative water content, PDM- Pod dry mass, RL- Root length, SL- Shoot length, RDM-Root dry mass and SDM- Shoot dry mass. (*) represent significant correlation:

*P≤ 0.05, and

**P ≤ 0.01.

In general, moderate correlations have been recorded between antioxidant enzymes with growth and other physio-biochemical parameters (**Table 1**). H_2_O_2_ and MDA depicted a moderately negative correlation with antioxidant enzymes. H_2_O_2_ was found negatively correlated with CAT (*r* = −0.690**), POD (*r* = −0.705**), APX (*r* = −0.633**) and GR (*r* = −0.415**) activities. These results indicate a strong participation of various ROS-scavenging enzymes in scavenging the H_2_O_2_ and thereby minimised membrane damage. RWC had a positive and statistically significant correlation with four (CAT, APX, GR and AsA) of the five antioxidant traits studied. Further, various other growth parameters were found moderately correlated with each other. The shoot dry mass (SD) and root dry mass (RD) showed significant positive correlation with other growth parameters. A positive correlation was also observed between root dry mass and pod mass (*r* = 0.658**) under DS, indicating that improved root trait in T lines contributed to enhancing the productivity under DS [8].

The enhanced tolerance under DS might be associated with stress-inducible expression of *AtDREB1A* gene in T peanut lines [8]. The expression of *AtDREB1A* in T peanut lines resulted in the up-regulation of downstream genes, responsible for biosynthesis of these antioxidant enzymes. On the similar note, up-regulation of antioxidant-producing genes under DS is also reported in tomato expressing *AtDREB1A* [56] and peanut expressing *mtlD* [63].

### Expression analysis of downstream abiotic stress-inducible genes

Expression analyses of downstream inducible genes of DREB regulon in various cops have helped in understanding the molecular mechanisms of signal transduction pathways involving various abiotic stress responses [73, 74]. The candidate genes selected for the expression studies using q-PCR were categorised into (1) Abiotic stress-inducible genes (*ABARP17-*ABA responsive protein abr17; *ERDEHYFP-* Early dehydration family protein; *LEAP*-Late-embryogenesis protein; *LEAE2*- Late embryogenesis abundant2), (2) Ion transporter gene (*AQAP1-ISO1*-Aaquaporin pip2–1-like isoform1, and (3) Membrane protein gene (*ANNEX1-*Aannexin) [43]. These target genes are known players in providing tolerance to different abiotic stresses, however, their precise role and cross-talk possibilities with other stress pathways are not yet deciphered [43].

**Fig 6:**
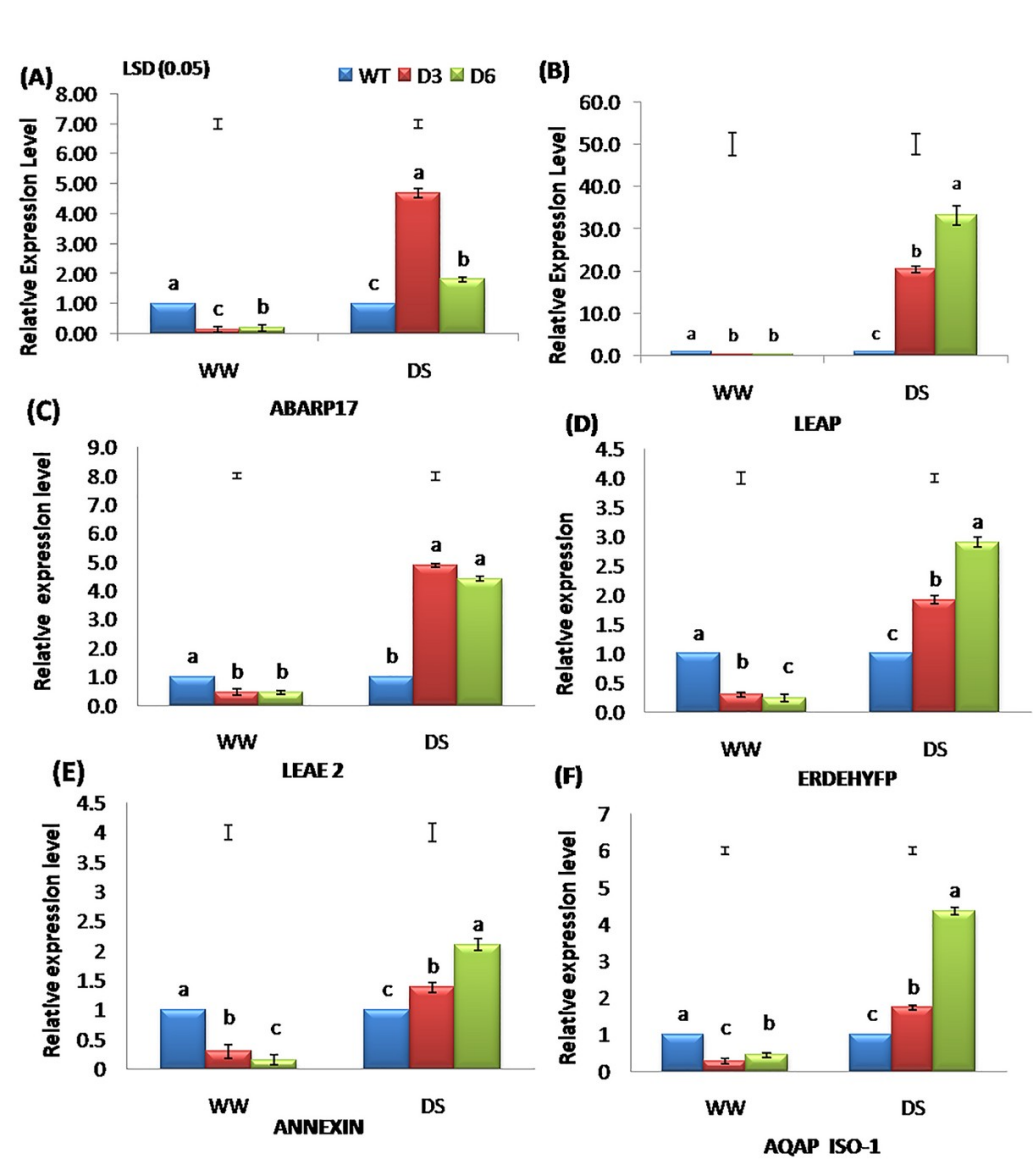
Relative fold change in (A) *ABARP17*, (B) *LEAP*, (C) *LEAE2*, (D) *ERDEHYFP*, (E) *ANNEXIN*, and (F) *AQAP* gene expressions in two selected *AtDREB1A* transgenic peanut lines at WW and after 25 days of DS imposition.

Abscisic acid induces leaf stomata closure to reduce transpiration, thereby improving water use efficiency (WUE) of plants [8, 75]. The *ABARP17* gene expression showed 4.69 and 1.81 fold increases in T lines D3 and D6, respectively over WT (**Fig 6A**). The promoter sequences of several genes, regulated by *AtDREB1A*, have a number of putative stress-related *cis*-acting elements, namely *DRE/CRT*, *MYBRS*, *MYCRS* and *ABRE*, which seem responsible for DS responsiveness [76]. The *DREB* gene mainly plays an important role in the ABA-independent pathways for stress tolerance by inducing the expression of various genes in plants. However, some *DRE*/*CRT* motifs themselves [77] or in combination with *ABRE* (abscisic acid response element) [78] can respond to an ABA-dependent pathway. The significant differences observed in *ABARP17* expressions between WT and T peanut plants might be indicative of *AtDREB1A*-regulated cross-talk between ABA-dependent and independent pathways [79].

The over-expressed *DREBs/CBFs* are known to enhance the expression of LEA gene [79] in response to drought and cold stresses, with an active expression in tissues containing high ABA [80]. Further, cross-talks have been noted between different abiotic stress signalling pathways during adaptation of plants.

The LEAP is known to act as a desiccation protectant and is associated with the protection of macromolecules, including enzymes and lipids [81,82]. The current study showed 20.39 and 33.05 fold expression of *LEAP* (**Fig 6B**), while *LEAE2* expressed 4.87 and 4.41 fold expression in D3 and D6 transgenic lines, respectively, over WT (**Fig 6C**). This high level of expression of LEA proteins in T lines might be responsible for the better retention of chlorophyll pigments and membrane stability than WT peanut as indicated in *ZmLEA3* and *AdLEA* tobacco lines [83, 82]. Early responsive to dehydration (*ERD*) genes gets rapidly activated during DS [84] and it triggers the programmed cell death signals and function as ‘connector’ for multiple stress response pathways [84]. Further, the *ERDEHYFP* gene expression analysis showed 1.91 and 2.90 fold increase in T lines D3 and D6, respectively, over WT under DS (**Fig 6D**). This increased expression in T lines indicates that the ERDEHYFP may act as a *connector* in the cross-talk among various signalling pathways under DS.

We also found up-regulation of the activity *ANNEX–1* gene by 1.37 and 2.09 folds in the T lines D3 and D6, respectively over WT (**Fig 6E**). Annexins are known to counteract the oxidative stress, maintain the cell redox homeostasis and retain the membrane stability, thereby imparting DS tolerance [85, 86]. Further, ectopic expression of Annexin gene of *Brassica juncea* has also been known to develop tolerance to abiotic stress in tobacco [85].

Aquaporin is known to transport water and other small molecules through cell membranes, and it also enhances plants’ ability to tolerate abiotic stress by improving WUE, hydraulic conductivity and by retaining better water status [87]. We observed a significant up-regulation of *AQAP1-ISO1* (aquaporin pip2-1-like isoform 1) gene with a fold change of up to 1.73 and 4.36 in T lines D3 and D6, respectively (**Fig 6F**). The improved cell water status, as indicated by the high level of RWC, might have resulted from up-regulation of *AQAP1-ISO1* gene by *AtDREB1A* gene in T lines under DS. The regulation of multiple aquaporin genes by *DREB* gene is also reported in *Arabidopsis* [87]. Furthermore, over-expression of a native aquaporin gene (*MusaPIP2;6*) in banana has also showed improved tolerance to salt and DS [88].

The qPCR assay of target genes provided evidence that *AtDREB1A* in T peanut acts as a ‘master regulator’ by modulating the expression of several stress-inducible genes including those involved in cross-talks among signalling pathways, water transport, molecular chaperone function (protection of cellular macromolecules) and detoxification under DS.

## Conclusion

DS has a profound and deleterious effect on the peanut yield, especially at reproductive stages when flowering, pegging and pod development occurs. In this study, eight stable *AtDREB1A* gene T peanut lines (D1–D8) were thoroughly characterised under soil-moisture deficit stress at reproductive stage, in lysimeteric system. Although, all the T lines showed improved antioxidant and physiological parameters but, D6 line outperformed others for most of the evaluation parameters. Thus, the line D6 could serve as a potential candidate cultivar for the drought-affected areas [8]. In addition, the T line D6, could also be used as valuable pre-breeding resource in back-cross breeding programmes to develop drought-tolerant, high-yield peanut varieties for commercial cultivation in drought-prone areas/hotspots, not only in India but across the world [13]. Moreover, the qPCR-based validation of stress-inducible and novel genes has opened new avenues in functional genomics and *cisgenic* approaches for the peanut crop. Also, the qPCR assay confirmed the cross-talk between ABA-dependent and independent signalling pathways through interactions with other stress-inducible proteins/TFs under DS. Thus, this investigation provides evidence that the heterologous expression of *AtDREB1A* gene in T peanut has led to an enhanced antioxidant mechanism, improved membrane stability, and higher chlorophyll retention capacity. Further, better cellular water status could be attributed to the efficient water-mining and conservation mechanisms, which are positively associated with improved terminal DS tolerance and growth-related parameters, including yield gain, under DS.

## Supporting information

S1 Table List of primers used for the differential expression of stress-responsive genes. (DOCX)
