# Supplementary material for: Regulation of antioxidant mechanisms by AtDREB1A improves soil-moisture deficit stress tolerance in transgenic peanut (Arachis hypogaea L.)
Source: PLoS One. 2019 May 9;14(5):e0216706. doi: 10.1371/journal.pone.0216706 (PMC6508701; doi:10.1371/journal.pone.0216706)
Supplement: S1 Table — (DOCX) [file pone.0216706.s001.docx]

**Table S1: List of primers used for the differential expression of stress-responsive genes.**

| **Genes** | **Sequences (5’-3’)** | **TA** (°C) |
| --- | --- | --- |
| ANNEX-1 | F -GGACTTCAAGCAATCAGGTTTT-  R -CTCCCCAGTTGTGTGATGAG- | 59.6  59.1 |
| *AQAP1-ISO1* | F -TGGTGAAGGACGTTGAGGTT-  R -GCAATCAAGGCTCTGTAGAAGG- | 59.16  59.09 |
| *ERDEHYFP* | F -TGAACTTGATGAGGAGAGTGG-  R -TGGAGAAACTAATGGTGTGGAG- | 57.03  57.71 |
| *LEAP* | F -TATGGCTCGTTCTTTCTCTGCTGT-  R -ATCTTGCCGCCGCTTATGGT- | 62.13  62.61 |
| *LEAE2* | F -AGGTGGGAGGAGGAAGAAGA-  R -CAGTGGTAGTGGTGGTGGTG- | 60.2  59.9 |
| *ABARP17* | F -ACTCTCACAACCCTCTTCACTC-  R -AGTTCATCGCCGTCTACCAC- | 59.37  59.83 |
